# Health Literacy and Frailty in Community-Dwelling Older Adults: Evidence from a Nationwide Cohort Study in South Korea

**DOI:** 10.3390/ijerph18157918

**Published:** 2021-07-27

**Authors:** Hye-Ri Shin, Eun-Young Choi, Su-Kyung Kim, Hee-Yun Lee, Young-Sun Kim

**Affiliations:** 1 Department of Gerontology, Graduate School of East-West Medical Science, Kyung Hee University, Yongin 17104, Korea; ltc.shinhyeri@gmail.com; 2 Leonard Davis School of Gerontology, University of Southern California, Los Angeles, CA 90089, USA; choieuny@usc.edu; 3 AgeTech-Service Convergence Major, Graduate School of East-West Medical Science, Kyung Hee University, Yongin 17104, Korea; godwithme2017@khu.ac.kr; 4 School of Social Work, The University of Alabama, Tuscaloosa, AL 35487, USA; hlee94@ua.edu

**Keywords:** health literacy, frailty, community-dwelling older adults, propensity score matching

## Abstract

Health literacy is closely associated with poor health outcomes and mortality. However, only a handful of studies have examined the association between health literacy and frailty status. The current study used data from a nationwide sample of Korean adults aged 70–84 collected from 10 cities, each of which represents a different region of South Korea (*n* = 1521). We used the propensity score matching (PSM) method to minimize the potential selection bias and confounding factors that are present in observational studies. After PSM, demographic and health-related characteristics between the limited health literacy (*n* = 486) and the nonlimited health literacy (*n* = 486) groups were not significantly different. Multinomial logistic regression analyses were conducted for the PSM-matched sample to examine the association between health literacy and frailty outcomes, where the robust group was set as a reference. Limited health literacy significantly increased the risk of pre-frailty (RRR = 1.45, *p* = 0.02) and frailty (RRR = 2.03, *p* = 0.01) after adjusting for demographic and health-related factors. Our findings underscore the need to foster health literacy programs and provide preliminary evidence to inform tailored intervention programs so that we might attenuate the risk of frailty in the older population.

## 1. Introduction

Frailty refers to a state of significant physiological decline with aging, and it is characterized by increased vulnerability to stressors and reduced functional reserves [1]. Frailty is associated with adverse health outcomes, including falls, hospitalization, poor quality of life, and mortality [2,3]. The Fried frailty phenotype [4] proposed the following five criteria for the diagnosis of frailty: (1) unintended weight loss, (2) fatigue, (3) grip strength, (4) reduced walking speed, and (5) decreased physical ability. People with three or more symptoms are identified as frail, and those with two symptoms are classified as being in the pre-frailty stage [4]. Previous studies found that frailty status is related to a wide range of sociodemographic characteristics (e.g., education and economic status), physical health conditions, and health behaviors [5,6], as well as psychological risk factors such as cognitive impairment, depression, and emotional frailty [7,8,9,10,11]. Continued research efforts are warranted to identify modifiable preventive and risk factors of frailty, which will contribute to its prevention and management. 

A recent growing body of health literature has focused on the role of health literacy. Health literacy is defined as one’s cognitive and social skills for reading, understanding, assessing, and using accurate health information to make informed health-related decisions and to promote good health [12]. Limited health literacy not only leads to poor recognition and bad management of one’s health condition, but also acts as a barrier to communication with health professionals and to access to medical treatment [13,14,15]. Systematic reviews have shown that health literacy is closely associated with poor health outcomes and mortality [16,17]. 

However, only a handful of studies have examined the relationship between health literacy and frailty status [18,19,20,21,22,23,24]. For example, in a study of 603 community-dwelling older adults in Taiwan [18], low health literacy was associated with higher odds of pre-frailty and frailty. The significance remained even after controlling for demographic and health covariates. Shah et al. surveyed 470 adults with an average age of 57 years, and found that those with high health literacy had a lower risk of frailty [21]. Similarly, according to a study conducted by Yamada et al. older adults with inadequate health literacy were disproportionately higher in the pre-frailty (52.2%) and frailty (65.7%) groups than they were in the healthy group (37.6%) [23]. 

Prior studies have provided some initial evidence for the significant role of health literacy in determining frailty status; however, they mainly used small, convenience samples recruited from geographically limited areas, thus limiting the representativeness of the findings. In addition, not all confounders, such as health behaviors, were adjusted in previous research models. Thus, it remains unclear whether health literacy leads to an increased risk of frailty among older adults. To address these research gaps, the current study used data collected from a large nationwide sample of Korean adults aged 70–84, from 10 study centers representing different regions of South Korea. We used the propensity score matching (PSM) method, which is known to minimize potential selection bias and confounding factors that are present in observational studies, and therefore, contributed to the improved internal validity of our findings [25]. 

## 2. Materials and Methods

### 2.1. Study Population

**Figure 1:**
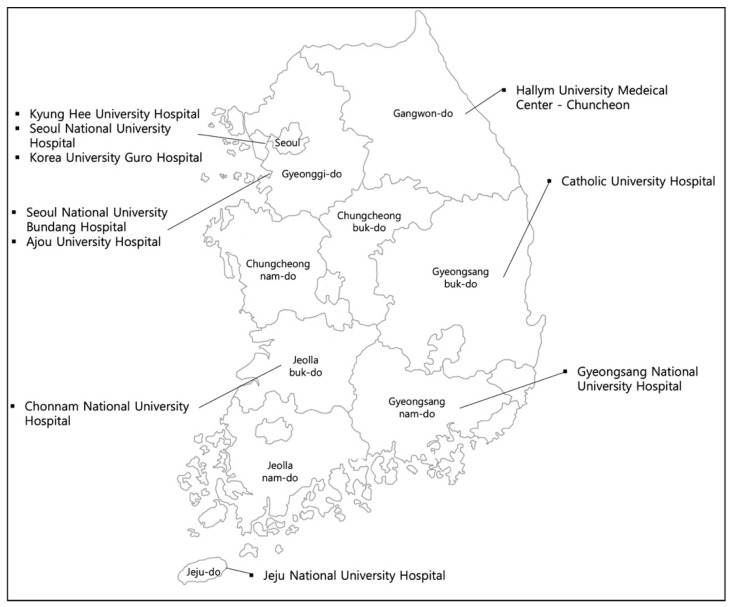
Ten study centers selected for the Korean Frailty and Aging Cohort Study (KFACS).

We used data from the Korean Frailty and Aging Cohort Study (KFACS), a population-based prospective cohort study of older Koreans [26]. The KFACS aims to evaluate and track older adults’ frailty status for 10 years and to identify relevant risk factors or outcomes. The baseline data were obtained between May 2016 and November 2017. As shown in Figure 1, ten study centers representing different regions of South Korea were chosen to increase the generalizability of the study and to address geographic variations. In each center, approximately 300 participants were recruited through quota sampling that was based on age and sex. The inclusion criteria for the study were as follows: (a) adults aged 70–84 years, (b) community-dwelling, (c) no plans to move out in two years, (d) no difficulties in communication, and (e) not diagnosed with dementia. Among a targeted total of 3000 people, half of the participants were recruited in 2016 (*n* = 1559) and the other half was surveyed in 2017 (*n* = 1455). The current study focused on those who completed the baseline interview in 2016. We excluded 38 respondents (2.43%) who had missing values in any variables of interest, resulting in a final analytic sample of 1521 participants.

### 2.2. Measurement

Frailty status was evaluated with the Korean frailty index (KFI), which was developed by a Korean Geriatrics Society research panel and previously validated among community-dwelling older adults [27,28]. The KFI consists of the following eight items: (a) a history of hospitalization in the past year (1 = yes), (b) self-rated health status (1 = poor), (c) weight loss in the past year to the extent that one’s clothes fit loosely (1 = yes), (d) polypharmacy (1 = taking four or more medications regularly), (e) depressed mood (1 = experiencing sadness or depressed mood for sometimes or more frequently in the last month), (f) incontinence (1 = experiencing incontinence of urine or feces for sometimes or more frequently in the last month), (g) visual or auditory problems (1 = any problems with decreased visual acuity or difficulties with hearing in daily life), and (h) physical performance measured with the Times Up and Go Test (TUGT) (1 = taking more than 10 seconds). Respondents received 1 point if they reported a positive response for each item. Those with two or fewer points were classified as robust; those with three or four points were considered as pre-frail; those with five or more points were categorized as a frail group.

Health literacy was assessed using the three questions adopted from the Behavioral Risk Factor Surveillance System (BRFSS) questionnaire, developed by the U.S. Centers for Disease Control and Prevention. The questions were as follows: (a) “How difficult is it for you to get advice about health or medical topics if you need it?” (b) “How difficult is it for you to understand information that doctors, nurses, and other health professionals tell you?” and (c) “You can find written information about health on the internet, in newspapers and magazines, and brochures in the doctor’s office and clinic; In general, how difficult is it for you to understand written health information?” The response options were “*very easy*,” “*somewhat easy*,” “*somewhat difficult*,” “*very difficult*,” and “*I do not look for health information*.” Respondents were categorized as having limited health literacy if they answered “*somewhat difficult*,” “*very difficult*,” or “*I do not look for health information*” to one or more questions, based on previous research [29]. 

We included the following sociodemographic characteristics and health-related factors as control variables: gender, age, marital status (married vs. single/divorced/widowed), living arrangements (living alone, living with a spouse only, or living with a spouse and/or children), income status (receiving basic livelihood security or medical benefits vs. not eligible), educational attainment (1 = no formal education, 2 = elementary school, 3 = middle school, 4 = high school, 5 = college or above), working status (yes vs. no), current smoking (yes vs. no), alcohol drinking (yes vs. no), and fall experience in the past year (yes vs. no). 

### 2.3. Statistical Analysis

The main purpose of the current study was to examine whether the limited health literacy and the nonlimited health literacy groups are at different risks of pre-frailty or frailty. As health literacy groups were not likely to be randomly assigned to our study population, we used the propensity score matching (PSM) method to account for potential selection and confounding biases (e.g., demographic characteristics both associated with health literacy and frailty status) [30]. The PSM method is known to reduce these biases [31], contributing to a more reasonable comparison between groups and improving the internal validity of the study findings. The strength of the PSM has broadened the range of research opportunities because it helps to analyze real-world data—nonrandomized data—with reduced selection bias [32]. However, there is criticism that PSM might be blind to the large portion of imbalance [33]. Therefore, caution is needed when conducting the PSM and interpreting the findings.

To create propensity score-matched pairs, we performed one-to-one matching using the Stata module of psmatch2 [34], where control variables of our study were used (gender, age, marital status, income status, education levels, working status, current smoking, alcohol drinking, and fall experience). The 486 nonlimited health literacy cases were matched with 486 limited health literacy cases, based on the nearest neighbor matching without replacement and conditioning on the common support. The pseudo-R2, an indicator of overall covariate imbalance, was lower after matching than it was before (after; pseudo-R2 = 0.002, mean bias = 2.1, median bias = 2.1, *p* = 0.997 vs. before; pseudo-R2 = 0.143, mean bias = 26.5, median bias = 22.8, *p* < 0.001). These findings suggest an adequate balance of covariate distribution among the matched groups. 

**Figure 2:**
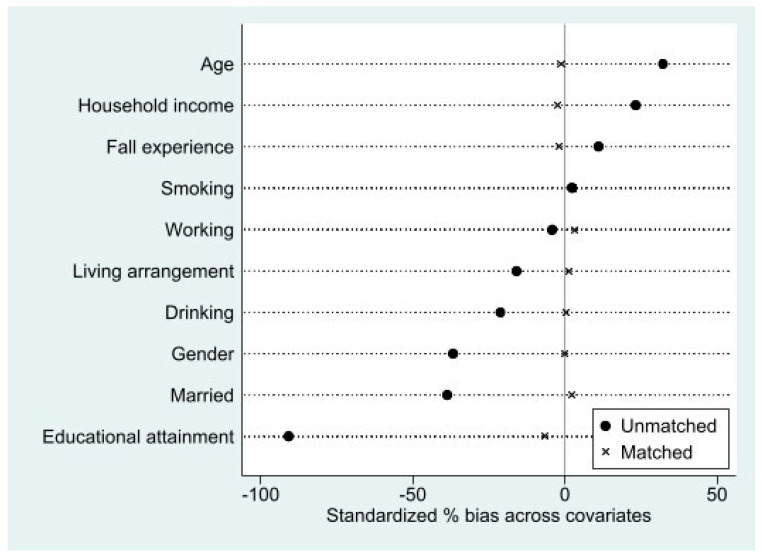
Visual inspection of standardized differences.

Figure 2 shows the standardized differences across covariates in the unmatched and matched samples. As expected, the standardized percent bias shifted toward 0 after matching. Then, we compared the limited and nonlimited health literacy groups in the full study sample (before the PSM; *n* = 1521) and the PS-matched sample (*n* = 972). Chi-square tests for categorical variables and *t*-tests for continuous variables were used to determine significant group differences. Finally, multinomial logistic regression analyses were conducted for the PSM-matched sample to examine the association between health literacy and frailty outcomes, where the robust group was set as a reference. All of the analyses were performed with Stata software version 16.0 (Stata Corporation, College Station, TX, USA). 

## 3. Results

### 3.1. Sample Characteristics of Limited and Nonlimited Health Literacy Groups 

Table 1 shows the descriptive characteristics between the limited health literacy (LHL) and nonlimited health literacy (Non-LHL) groups. Of the full study sample (*n* = 1521), approximately 68% had limited health literacy (*n* = 1035). Those in the LHL group were older (76.55), less likely to be married (59%), more likely to live alone (29%), have low household income (9%), and reported lower educational attainment (2.35) than the Non-LHL group. These demographic differences were statistically significant (Table 1). In addition, those in the LHL group were less likely to be drinking (46%) and more likely to have fall experience (22%) compared to the Non-LHL group. The results for both groups differed significantly (Table 1). The two groups also differed in their frailty status. Approximately 32% of the LHL group fell in the pre-frail category and 16% were in the frail category, which was significantly more than the 21% pre-frail and 5% frail in the Non-LHL group. After propensity score matching, the demographic and health-related characteristics between the LHL (*n* = 486) and Non-LHL (*n* = 486) groups were no longer significantly different. However, the LHL group still included more pre-frail (26%) and frail (8%) individuals than the Non-LHL group (Table 1).

### 3.2. Relative Risk Ratio of Pre-Frailty and Frailty 

Table 2 shows the relative risk ratios (RRRs) for the risk of pre-frailty and frailty in the propensity-matched sample (*n* = 972). We first conducted multinomial logistic regression analyses with a limited health literacy variable only to reduce the potential confounding effects by the treatment variables [35]. 

After adjusting for demographic and health-related factors, limited health literacy significantly increased the risk of pre-frailty (RRR = 1.45, 95% CI = 1.06–1.98, *p* = 0.02) and frailty (RRR = 2.03, 95% CI = 1.19–3.49, *p* = 0.01). Concerning the covariates, the risk of pre-frailty was significantly associated with age (RRR = 1.07, 95% CI = 1.03–1.12, *p* < 0.001), educational attainment (RRR = 0.81, 95% CI = 0.71–0.94, *p* = 0.01), and fall experience (RRR = 2.01, 95% CI = 1.37–2.95, *p* < 0.001). The risk of frailty was significantly increased by being male (RRR = 2.62, 95% CI = 1.30–5.26, *p* = 0.01) and being of an older age (RRR = 1.10, 95% CI = 1.02–1.18, *p* = 0.01), but decreased by living with a spouse (RRR = 0.15, 95% CI = 0.03–0.64, *p* = 0.01), living with a spouse and/or children (RRR = 0.10, 95% CI = 0.02–0.43, *p* < 0.001), having higher educational attainment (RRR = 0.68, 95% CI = 0.53–0.88, *p* < 0.001), and working (RRR = 0.49, 95% CI = 0.25–0.97, *p* = 0.04).

### 3.3. Sensitivity Analyses

We also examined the association between frailty status and health literacy among the full study sample. As presented in Table 3, the findings remained similar to the propensity-matched sample. Limited health literacy was related to an increased risk of both pre-frailty (RRR = 1.50, 95% CI = 1.13–2.00, *p* = 0.01) and frailty (RRR = 2.31, 95% CI = 1.43–3.73, *p* < 0.001). A few differences were found in the associations between gender, living arrangements, and fall experience in relation to frailty status. In the full sample, being male was not significantly associated with an increased risk of frailty (RRR = 1.06, 95% CI = 0.69–1.62, *p* = 0.81), whereas living with a spouse compared to living alone did not itself decrease the risk of frailty (RRR = 0.72, 95% CI = 0.34–1.55, *p* = 0.40). Fall experience, however, was significantly related to an increased risk of frailty (RRR = 2.17, 95% CI = 1.49–3.17, *p* < 0.001).

## 4. Discussion

As of 2017, South Korea became an older society in which adults aged 65 and above accounted for over 14% of the total population. By 2025, the nation is expected to become a super-aged society, as the ratio increases to 20.3% [36]. Older Koreans have approximately 2.7 chronic diseases on average [37]. Approximately 47% and 8% are estimated to have pre-frailty and frailty, respectively [26,28]. In light of this social context, there is an increasing need to identify preventive and risk factors of pre-frailty and frailty [7,8]. The current study focused on the role of health literacy in determining frailty status among community-dwelling older adults in South Korea. 

Our analyses with a nationwide sample found that approximately 32% and 16% of participants with limited health literacy (LHL) fell into the categories of pre-frail and frail, respectively. These rates were significantly higher compared to the 21% of pre-frailty and 5% of frailty in the nonlimited health literacy (Non-LHL) group. The findings from the multiple logistic regression showed that LHL significantly increased the risk of pre-frailty and frailty, even after a propensity score matching for demographic and other health factors. These findings are consistent with previous evidence [18,21,22,23], suggesting that a health literacy education and intervention program can be an effective strategy to prevent and decrease the risk of frailty. Indeed, systematic research has suggested that improving health literacy can facilitate subsequent health behavior changes, such as better diet quality, increased physical activity, and smoking cessation [16,38]. A recent intervention study targeting older adults with low health literacy [39] found that an active learning program is effective in promoting a healthier lifestyle (i.e., moderate-to-vigorous physical activity and dietary variety), which is itself a known protective factor against the development of frailty [40]. 

Furthermore, we identified different sets of demographic and health-related predictors of pre-frailty and frailty. In line with prior studies [41,42], age and educational attainment were significantly associated with the risk of pre-frailty and frailty. Fall experience had a significant relationship with pre-frailty status only, whereas being male, living alone, and nonworking status were related to an increased risk of frailty. It was notable that factors related to social activities (i.e., living arrangements and working status) were associated with frailty status (vs. robust). These findings generally follow the literature reporting several risk factors of pre-frailty and frailty [43,44,45]. Therefore, when planning intervention programs, more attention should be paid to vulnerable groups with limited sociodemographic data. For example, interventions for older adults living alone should involve a connection to local community centers, where a variety type of preventative programs for frailty can be provided. 

Some major strengths of our study include the use of a nationwide sample of older Koreans collected from 10 representative cities/provinces of South Korea; this approach addresses the paucity of research on this topic with a large sample of older adults. Additionally, we accounted for potential selection effects and confounding biases between health literacy groups through the PSM method. To the best of our knowledge, this study is the first to examine the role of limited health literacy in frailty outcomes using the PSM method.

It is important to note some limitations of our study when interpreting the findings. The cross-sectional design did not allow us to establish a direct causal relationship between independent variables and frailty. Second, we did not measure participants’ previous experiences with health-related education or programs, which may have influenced the association between health literacy level and frailty status. Lastly, future studies need to address the overmatching problem more carefully by conducting the robustness checks. Future studies would benefit from employing a more comprehensive range of control variables to further clarify the positive effects of health literacy in preventing frailty. 

## 5. Conclusions

The current study showed that limited health literacy is associated with an increased risk of pre-frailty and frailty among community-dwelling older adults. Our findings not only underscore the need to foster health literacy intervention programs, but also to provide preliminary evidence to inform tailored intervention programs by demographic and health-related factors to attenuate the risk of frailty in the older population.

## Figures and Tables

**Table 1:** Sample characteristics of the full study sample and propensity-matched sample.

	Full Study Sample (*n* = 1521)					Matched Sample (*n* = 972)				
	LHL (*n* = 1035)		Non-LHL (*n* = 486)			LHL (*n* = 486)		Non-LHL (*n* = 486)		
	%	Mean	%	Mean	*p*-Value	%	Mean	%	Mean	*p*-Value
**Demographics**										
Male	41.2		40.9			40.9		40.9		
Age		76.55		75.32	<0.001		75.25		75.32	0.79
Married	59.1		76.5		<0.001	78.2		76.5		0.54
Living arrangement										
Living alone	29.4		16.3		0.005	16.0		16.3		0.69
With a spouse only	44.8		60.1			58.8		60.1		
With a spouse and/or children	25.8		23.7			25.1		23.7		
Household income										
Low	9.0		3.5		<0.001	2.9		3.5		0.58
Middle and upper	91.0		96.5			97.1		96.5		
Educational attainment		2.35	0.0	3.50	<0.001		3.42	0.0	3.50	0.26
**Health-related factors**										
Working	25.2		26.7		0.52	28.0		26.7		0.67
Smoking	5.5		5.1		0.77	6.2		5.1		0.49
Drinking	46.2		56.6		<0.001	57.4		56.6		0.80
Fall experience	21.6		17.3		0.049	16.0		17.3		0.61
**Frailty status**										
Robust	52.0		74.3		<0.001	65.6		74.3		0.002
Pre-frail	31.9		20.6			25.9		20.6		
Frail	16.1		5.1			8.4		5.1		

Note. LHL = limited health literacy; to make comparisons between the health literacy groups, *t*-test was conducted for continuous variables and chi-square statistics were computed for categorical factors.

**Table 2:** Relative ratio of pre-frailty and frailty among propensity-score-matched sample (*n* = 972).

	Pre-Frail (*n* = 226)				Frail (*n* = 66)			
	vs. Robust (*n* = 680)				vs. Robust (*n* = 680)			
	RRR	95% CI		*p*-Value	RRR	95% CI		*p*-Value
**Main variable**								
Limited health literacy	2.21	1.70	2.87	<0.001	4.48	2.88	6.96	<0.001
**Main variable**								
Limited health literacy	1.45	1.06	1.98	0.02	2.03	1.19	3.49	0.01
**Demographics**								
Male	0.76	0.52	1.12	0.17	2.62	1.30	5.26	0.01
Age	1.07	1.03	1.12	<0.001	1.10	1.02	1.18	0.01
Married	1.19	0.60	2.34	0.62	2.95	0.66	13.13	0.16
Living arrangement (Ref = alone)								
With a spouse only	0.90	0.42	1.93	0.78	0.15	0.03	0.64	0.01
With a spouse and/or children	0.72	0.37	1.38	0.32	0.10	0.02	0.43	<0.001
Low income	1.31	0.53	3.24	0.56	1.73	0.54	5.53	0.35
Educational attainment	0.81	0.71	0.94	0.01	0.68	0.53	0.88	<0.001
**Health-related factors**								
Working	0.85	0.59	1.22	0.38	0.49	0.25	0.97	0.04
Smoking	1.22	0.61	2.44	0.57	1.21	0.46	3.18	0.70
Drinking	0.92	0.67	1.28	0.64	0.60	0.35	1.03	0.07
Fall experience	2.01	1.37	2.95	<0.001	1.71	0.89	3.29	0.11

Note. RRR = relative risk ratio.

**Table 3:** Relative risk ratio of pre-frailty and frailty among the full study sample (*n* = 1521).

	Pre-Frail (*n* = 430)				Frail (*n* = 192)			
	vs. Robust (*n* = 899)				vs. Robust (*n* = 899)			
	RRR	95% CI		*p*-Value	RRR	95% CI		*p*-Value
**Main variable**								
Limited health literacy	2.22	1.71	2.87	<0.001	4.66	3.00	7.24	<0.001
**Main variable**								
Limited health literacy	1.50	1.13	2.00	0.01	2.31	1.43	3.73	<0.001
**Demographics**								
Male	0.76	0.56	1.02	0.07	1.06	0.69	1.62	0.81
Age	1.08	1.05	1.12	<0.001	1.14	1.09	1.19	<0.001
Married	0.94	0.57	1.55	0.82	1.20	0.59	2.41	0.61
Living arrangement (Ref = alone)								
With a spouse only	1.01	0.59	1.75	0.96	0.72	0.34	1.55	0.40
With a spouse and/or children	0.68	0.45	1.04	0.07	0.55	0.31	0.98	0.04
Low income	1.20	0.75	1.93	0.45	1.15	0.64	2.09	0.64
Educational attainment	0.78	0.71	0.87	<0.001	0.64	0.55	0.75	<0.001
**Health-related factors**								
Working	0.83	0.63	1.11	0.21	0.50	0.32	0.77	<0.001
Smoking	0.97	0.54	1.74	0.93	1.78	0.92	3.44	0.09
Drinking	0.95	0.74	1.22	0.67	0.74	0.52	1.05	0.09
Fall experience	1.48	1.10	2.00	0.01	2.17	1.49	3.17	<0.001

Note. RRR = relative risk ratio.
